# "Three Nooses on Our Head": The Influence of District Health Reforms on Maternal Health Service Delivery in Vietnam

**DOI:** 10.15171/ijhpm.2017.134

**Published:** 2017-11-22

**Authors:** Nguyen Thi Hoai Thu, Fiona McDonald, Sophie Witter, Andrew Wilson

**Affiliations:** ^1^Institute for Preventive Medicine and Public Health, Hanoi Medical University, Hanoi, Vietnam.; ^2^Australian Centre for Health Law Research, Faculty of Law, Queensland University of Technology, Brisbane, QLD, Australia.; ^3^Institute for Global Health and Development, Queen Margaret University, Musselburgh, UK.; ^4^Menzies Centre for Health Policy, University of Sydney, Sydney, NSW, Australia.

**Keywords:** District Health Reforms, Health System Governance, Health Policy, Fragmentation of Services, Maternal Health, Vietnam

## Abstract

**Background:** The impact of reorganisation on health services delivery is a recurring issue in every healthcare system. In 2005 Vietnam reorganised the delivery of health services at the district level by splitting preventive, curative, and administrative roles. This qualitative study explored how these reforms impacted on the organisation of maternal health service delivery at district and commune levels.

**Methods:** Forty-three semi-structured interviews were conducted with health staff and managers involved in the provision of maternal health services from the commune to the central level within five districts of two Northern provinces in Vietnam. The data were analysed thematically.

**Results:** The results showed that 10 years after the reforms created three district-level entities, participants reported difficulties in management of health services at the district and commune levels in Vietnam. The reforms were largely perceived to negatively affect the efficient and effective use of clinical and other resources. At the commune level, the reforms are said to have affected the quality of supervision of the communes and their staff and increased the workload in community health centres.

**Conclusion:** The findings from this study suggest that the current organisation of district health services in Vietnam may have had unintended negative consequences. It also indicates that countries which decide to reform their systems in a manner similar to Vietnam need to pay attention to coordination between a multiplicity of agencies at the district level.

## Background

Ensuring the delivery of health services that are accessible, equitable, safe, and responsive to the needs of the population is considered the main objective of a health system.^1^ In the global health context, particularly in developing countries, strengthening health services has been recognised as a priority to meet the basic health needs of populations.^2^ Improving health service delivery is an essential part of strengthening health systems and requires context specific adaptions.^3^ One ongoing controversy is the tension between centralised and decentralised organisation and management of the health system.^4-6^ Another relates to the balance between vertical and horizontal programming and the challenge of integration across programs.^7^ These two issues are frequently inter-related and their impact on services, staff and patients depend on factors such as the manner of implementation, management capability, the extent of organisational, administrative and resource changes and the level of preparation of staff for change.^8^

Health sector reforms include a wide range of actions, however “Health sector reforms implies more than just any improvement in health or healthcare.”^9^ Over the past few decades, health sector reforms in many low- and middle-income countries have had a profound impact on the financing and organization of the health sector. Some countries have reported positive outcomes of reforms to the health sector. For example, in Kenya health sector reforms were considered to a have positive influence on the health system, establishing fiscally significant cost recovery with some equity protections in a very difficult environment.^9^ However, in some countries reforms have been reported to increase inequalities in access and utilization of health services, and/or having reduced public expenditure on the health sector.^9,10^ For example, Argentinian healthcare financing reforms have been criticised and has been reported as worsening the pre-existing weaknesses of the health sector, including its inefficiency.^11^

Health sector reforms can take many forms. Some reforms have been designed to change which organisations or institutions deliver types of health services,^12^ and hence have seen the fragmentation of health systems at various levels.^10,13^ Fragmentation is defined as “lack of coordination between the different levels and settings of care, duplication of services and infrastructure, unutilized productive capacity, and healthcare provided at the least appropriate location, especially hospitals.”^13^ The literature has indicated that fragmentation can lead to difficulties in access to services, delivery of services of poor technical quality, irrational and inefficient use of resources, unnecessary increases in production costs, and low user satisfaction.^1,14^ For example, the fragmentation of health insurance pools in the Chilean health system was reported as inefficient and has decreased solidarity between rich and poor, sick and healthy, and young and old.^15^ Fragmentation is not only of concern from an equity perspective, but also in relation to the efficiency and affordability of the health system.^10,12^ Little is known about the impact of fragmentation of health systems on the way that healthcare services at the grass root level in Vietnam are organised and operated and what lessons can be learnt internationally about the consequences of these reforms. Maternity services are provided at all levels interdependently across the Vietnamese health system and consequently are a good model to examine the impact of health service reorganisation. This study aimed to answer two questions: (1) How district health reforms have influenced the organisation of maternal health services? (2) Could governance mechanisms be strengthened to improve the organisation of maternal health services delivery?

**Figure 1:**
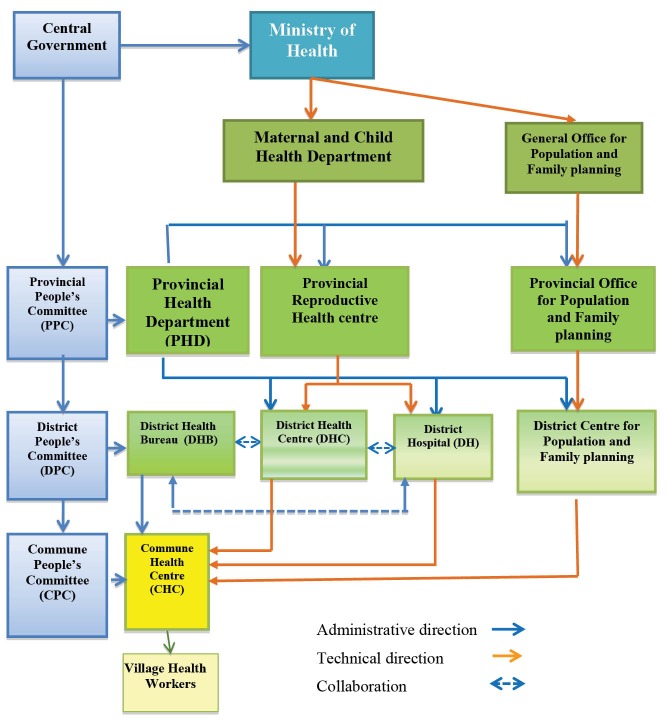
Organisation of the Vietnamese Healthcare System.
Sources: Adopted and modified from Report of Hanoi School of Public Health.^17^

Emerging economy countries like Vietnam face governance challenges in delivering quality and equitable health services.^16^ Publicly funded health services in Vietnam are provided at 4 levels. Primary care is offered through commune health centres (CHCs) in each commune (sub-district). Secondary hospital services are provided at the district level. Each province is divided into approximately 20 districts. Tertiary level hospital facilities are provided at the provincial or national level. See Figure 1.

**Figure 2:**
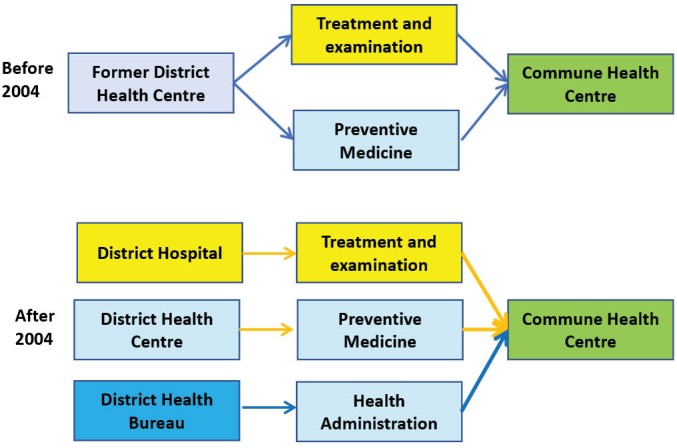
The Change in Functions of District Health Units After Implementing Reforms.

Before 2004, District Health Centres were the only entities in charge of all health programs and activities within a district. Former District Health Centres had curative and preventive functions and were responsible for providing healthcare within a district. Decrees 171 and 172 issued in 2004,^18^ which were lately replaced by Decrees 13 and 14 and subsequent policy circulars in 2008,^19-21^ provided the basis for a substantial reorganisation of district health services in 2005 and 2006. When these policies were implemented, new independent entities with separate functions (public administration; curative; preventative; and family planning) were created. The purpose of these reforms was to strengthen capacity in respect of each function, improve efficiency, service delivery quality, which in turn would improve the health status of the population.^16^ Figure 2 describes the changes in functions and responsibilities of district health units.

There was no specific requirement from the government for the districts to be reformed. The Decree regulated and instructed the reforms, but stated that the Provincial People’s Council should consider implementing the reforms if they considered them suited to the current context of the province. As a result, a number of provinces delayed or did not implement the reform process. In those provinces that instituted the reforms, depended on conditions in each locality, the delivery of health services at the district level was organised with a District Health Centre, District Hospital and a District Centre for Population and Family Planning.^16^ Some localities have not yet implemented the reforms, maintaining the old model of an integrated District Health Centres plus the District Hospitals. This led to a situation where there are multiple organisational models at the district level in different provinces, or even between districts within the same province. Vietnam presently has 697 districts of whom 460 have instituted the reforms and 233 have not, while other districts have not yet reported.^22^

District Health Bureaus are responsible to the District People’s Committees and advise the District People’s Committees on the management of CHCs and perform designated tasks as authorised by the District People’s Committees and the Provincial Health Department. However, in some places CHCs may be supervised by the District Health Bureau or the District Health Centre. The District People’s Committees control the District Health Bureaus in terms of their direction, organisational management, payroll, and operations, whereas the Provincial Health Department provides technical direction, guidance, monitoring, and inspection.

District Hospitals were split from the former District Health Centres and are now under the stewardship and management of the Provincial Health Department. District Hospitals provide diagnostic and therapeutic (curative) services to the local population and serve as the first referral point for CHCs within the district.

The post-reform District Health Centres now have a largely preventative focus with responsibility for national health target programs including reproductive health programs. District Health Centres supervise CHCs for all vertical programs and, in many provinces, supervise all CHC staff.

District Centres for Population and Family Planning are under the supervision of the provincial Population and Family Planning Branch. The Centres are responsible for technical tasks associated with family planning, and communication and education about population and family planning within the district.

CHCs provide comprehensive primary healthcare at the commune level. They provide care and treatment for common diseases, conduct normal deliveries, promote family planning and preventive hygiene and offer health promotion programs. The administrative management of the CHCs are the responsibility of the District Health Bureaus and the Commune People’s Committees, with technical support from the District Hospital and the District Health Centre. A CHC may serve from 2000-10 000 people, depending on the region or levels of deprivation. Village health workers are responsible for primary healthcare functions, including healthcare for mothers and children at the village level.^23^

## Methods

This paper reports one part of a broader study that took place in 5 rural districts in Bac Giang and Lao Cai. Lao Cai is a province in the northern mountainous area of Vietnam, located around 320 km northwest of Hanoi. Bac Giang is in the northern midlands and mountainous area of Vietnam, being situated 50 km to the east of Hanoi. The two provinces were chosen based on the indicators of maternal health services reported to the Ministry of Health. Lao Cai had relatively low performance on the indicators for this output, while Bac Giang had much higher indicators, which include the proportion of women having at least 3 antenatal check-ups and the proportion of women attended by skilled staff. Both provinces had generally instituted the district governance reforms, although one province did not transfer the responsibility for the supervision of CHCs from the District Health Bureau to the District Health Centre. The five districts were purposively selected as typical rural districts in Northern Vietnam, with one province having a significant ethnic population.

A qualitative approach was used in order to allow an in-depth exploration of factors impacting on organisation of maternal health services in Vietnam. Participants included health workers from the commune and district levels and health managers from all four levels of healthcare system in Vietnam. These participants were purposively selected because of their roles in the provision or management of maternal health services.^24^ At the central and provincial levels, key informants were selected based on their responsibilities and functions. At the district and commune levels, apart from purposive sampling, the approach also involved the “snow ball or chain” approach, with the number of interviews determined by the point that responses to particular questions are saturated, that is, no new information is being added.^25^ Forty-three semi-structured interviews were conducted. Table describes the characteristics of the participants.

**Table:** Description of Participants

	**No. (%)**
Gender	
Male	16 (37.2)
Female	27 (62.8)
Age	
≤30	4 ( 9.3)
31-40	7 (16.3)
41-50	24 (55.8)
>50	8 (18.6)
Work experience (y)	
≤10	7 (16.3)
11-20	23 (53.5)
21-30	8 (18.6)
>30	5 (11.6)
Qualification	
Secondary	14 (32.6)
University and College	9 (20.9)
Post graduate	20 (46.5)
Level in the health system	
Central	2 (4.7 )
Provincial	6 (14.0)
District	26 (60.4)
Commune	9 (20.9)
Type of facility	
Commune Health Centre	9 (20.9)
District Hospital	17 (39.5)
District Health Centre	9 (20.9)
Reproductive Health Centre	4 ( 9.3)
District Health Bureau	2 (4.7 )
Provincial Health Department	2 (4.7 )
Total	43

Interview guides were developed and piloted with health staff and managers before using them to guide the interview. The interviews aimed to explore the transformation process of district health system from the old model into the three district health entity model, and the roles and functions of district health entities in the supervision of CHCs’ performance. The findings of these interviews were used to further develop the conceptual framework and to guide the development of tools in the main study.

Interviews were conducted in Vietnamese by the lead author at the health worker’s or health manager’s place of work in a private space. Each interview took approximately 60 minutes. All interviews were audio-recorded, transcribed, checked for quality and translated into English. A number of key documents were also purposively selected and reviewed.

All transcripts were uploaded into NVivo 9.0^26^ and single coded by the principal researcher. First, the transcripts of these interviews were independently scrutinised and coded manually. An initial phase of coding on three transcriptions of representative respondents for commune, district and provincial levels were conducted by the primary researcher and research team members, using the preliminary coding schedule and also applying grounded theory techniques^27^ with quotations servings as units of analysis.^24^ This approach provided a way of synthesising data, developing concepts and also testing emergent concepts with additional fieldwork^24^ and helped identify open codes that were not part of the conceptual framework. The results of the parallel coding process were then compared, validated and agreed in order to form the initial code book. This process of developing the initial code book also involved revisiting the summaries of field notes^28,29^ and the existing literature review, in particular in relation to the categories set out in the conceptual framework. Then, the final code book of major codes with the definition for each of those was developed. Subsequently a consensus coding structure reflecting core themes was developed through research team discussions.

Throughout this process, coding was reviewed for consistency, revised and refined in an effort to link the core themes to the research questions.^30^ The open codes were grouped in meaningful patterns or dimensions, thereafter creating categories. Categories are subsequently re-organised and linked together in order to understand the relationships among categories, highlight patterns and clarify interpretation. The whole process of “making sense of data” was linked to the literature review.^28^ The initial findings were written up by the principal researcher and shared with research team members. The validation and interpretation of data was undertaken against the quotes from transcripts. Memos and field notes were used to help triangulate interview data.

The policy documents related to district health reforms were retrieved and analysed, focusing on the functions, responsibilities and coordination between the new entities. In addition, reports from provincial health departments and district health entities were collected and analysed to provide information related to task division and collaboration of entities in the organisation of maternal health service delivery.

## Results

This research provides evidence of how fragmentation in health systems occurred when reforms to district level governance saw a move from an integrated model to a model where functions were disaggregated. This section will display the difficulties perceived by district level respondents in collaborating with other units and in the managing of CHCs, and by commune level respondents in performing their assigned tasks.

### District Level

#### Dilution in Human Resources

In respect of human resource management, the analysis identified two key issues: the exacerbation of workforce shortages; and lack of management skills. The creation of multiple entities at the district level has the potential to exacerbate the existing shortages of qualified health workers. Each district health entity should have at least one director and one or two deputy directors who usually have the highest qualifications amongst the health workers in the centre. For example, in a Centre for Population and Family Planning, there are 6-7 health workers, one of whom acts as the Centre director and another health worker who acts as a budget officer. The separation of functions at the district health level unintentionally appears to have weakened the internal capacity of the district health system as it requires many more of the better clinically qualified and competent health workers to assume management roles. One participant noted:

“*Splitting the former district health centre into different health units requires the promotion of many staff to managerial positions. The consequence is that we lack people who can conduct the clinical work, it is just a waste of resource*s” (District Level, Manager 12).

The district health entities may not have the resources to recruit additional clinical staff to fill the clinical roles formerly undertaken by managers. As a consequence managers may feel pressure to undertake managerial and clinical roles.

There was also concern expressed about the impact of the elevation of health workers to managerial roles without training in human resource management. Several district managers expressed the view that they were not able to evaluate health workers’ performance because they did not have human resource management skills. This in turn was said to result in loss of job satisfaction, as managers were not satisfied with their current management skills and due to their management role also could not contribute significantly to the clinical areas in which they were trained. It was claimed that the district health system gradually had been losing the “good clinical performers” in exchange for “ineffective managers.” It was also argued that the lack of management skills negatively impacted upon health workers under their supervision.

#### Lack of Clarity of Responsibilities of District Health Entities

While the structure of the system in Lao Cai province suggests that the District Health Bureaus are responsible for the supervision of the personnel and administrative processes of the CHCs, the District Health Centre has technical supervision of CHCs in respect of implementing target programs and maternal health programs, and the District Hospital supervises CHCs in diagnostic and therapeutic services, in practice the actual responsibilities of these entities are less clear.

The District Health Bureau’s role in both study locations was differently understood by respondents. Respondents working in the District Health Bureau in both provinces thought that their function at a district level was similar to the function of the Provincial Health Department at the provincial level, which is to manage all health-related activities in the area.

*“The District Health Bureau, if understood correctly, is the unit that manages all health activities in the district, not only CHCs. But we do not have enough personnel and budget so our voice is not appreciated. This is really a problem in health sector” (*District level, Manager 2).

By contrast, other participants from the District Hospitals and District Health Centres viewed District Health Bureaus as administrative units that do not implement any specific health programs, and that its main function is to assist the District People’s Committees and manage private medical practice within the district:

*“...they [District Health Bureaus] are only one department under the District Peoples Committee with function to provide advice to health issues in the district. However, they overplay their role, so it sometimes is very bad” (*District level, Manager 12).

More importantly, the District Hospital and District Health Centre managers considered their organisation to be responsible for direct implementation of a program, not the District Health Bureau. There seemed to be a disagreement as to the supervisory responsibilities of district health entities that reportedly caused delays in many processes at the CHC.

The District Health Bureaus, District Hospitals, and District Health Centres all had a role in supervision of CHCs but the parameters of their responsibility were unclear, resulting in inconsistencies and ineffective or inefficient supervision. The leading and supporting roles in curative care of the District Hospital towards the CHCs has declined, because the CHCs are directly under the management of the District Health Centre.^16^ For example, the District Hospital may have the responsibility to supervise CHCs in respect of treatment and examination, but in practice they supervised the assistant doctors or medical doctors who performed some examinations in CHCs. The District Hospital did not supervise the midwife who performs deliveries, as maternal health is considered a preventative service and is supervised by the District Health Centre.

The District Health Centre, with its primary responsibility being to implement preventive programmes, usually does not have much capacity to provide professional mentoring and supervision of curative care activities of the CHCs, an important part of primary healthcare. There was common consensus among respondents that with limited competencies, many health workers in District Health Centre’s are not able to direct CHCs in the maternal health area.

*“Our health workforce do not have obstetrics expertise so they need some short training courses on reproductive health area, however it is not as good as those have obstetrics expertise. Therefore we have problem in supervision. They are at district level and responsible to direct CHCs. If they have expertise, the technical supervision for CHCs must be better”* (Provincial level, Administrator 3).

#### Lack of Collaborative Mechanisms Among Three District Health Entities

A common theme emerging from the interviews was the poor information transmission processes between entities, described by one participant as a “zigzag line” which requires a considerable amount of time to navigate.

*“I think that the point is the organisational structure and management mechanisms. In the past only one district health centre was in charge of both curative and preventive functions, including development of vertical programs, reproductive health services and population programs. The additional function was to advise the District People’s Committee on delivering health administration. In short, the Provincial Health Department or any other provincial bodies, any departments of the District Peoples Committees worked only with leaders of district health centres. Now if you want to undertake some work, you have to go through a zigzag line. It depends what responsibility belongs to which unit, hospital or district health centre, or health bureau or centre for family planning and population. Oh, it’s very time consuming, but that still does not take disunity into account”* (District level, Manager 13).

Since the separation of responsibilities in 2004, the District Health Bureau reports to the District People’s Committee, while the District Hospital and the District Health Centre are supervised by the Provincial Health Department. For some services, all three draw on the same CHC resources and CHCs report to the District Health Bureau. This means the District Hospital and the District Health Centre need to negotiate first with the District Health Bureau, and then approval must be sought from the District People’s Committee before the District Hospital and the District Health Centre can implement programs at CHCs. It was reported by participants that prior to the reforms all health-related activities were managed by the then District Health Centre and the Centre was proactive in cooperating with other health facilities within the district and the District People’s Committee. As a consequence, the health sector was able to mobilise more resources, and health-related activities were developed in a timely and effective way. The pre-reform District Health Centre had the flexibility to deploy and rotate health workers within the Centre in order to implement any program, particularly in an emergency or in respect of epidemic prevention.

After the reforms, the unclear responsibilities of the District Health Bureau, coupled with a lack of effective cooperation between entities, have reportedly led to inconsistencies between the different entities in program implementation.

*“The District Hospital considers that the District Health Bureau is not so important, so sometimes they do not discuss with us about the activities to be implemented at CHCs such as examination and treatment, drug and medical equipment supplies. They just go directly to CHCs to run their programs. When we received the report, all of the activities have already been developed” (*District level, Manager 2).

Ill-defined responsibilities, divided loyalties and a lack of clear cooperation mechanisms were reported to result in dysfunctional joint meetings. With three district health entities considered to have equal power, collaboration was thought to be affected by the unit leaders’ attitudes.

*“The regulation on collaboration may exist, but if they are not unified and collaborative, he [a leader of one district health unit] can refuse my invitation to the meeting because I am not his boss. But if the chair of District Peoples Committee or leader of Provincial Health Department calls the meeting, I obviously have to go” (*District level, Manager 13).

Participants argued that there needed to be strict regulation to force district health entities to collaborate effectively.

*“If each of them considers themselves powerful, it is very difficult to work together”* (District level, Manager 13).

The next section will discuss the impact of the poor coordination among district health entities (District Health Bureau, District Hospital and District Health Centre) on oversight of the CHCs.

### Commune Level

The creation of the independent district health entities in 2005 in the study provinces was said to have had an impact on the operations of the CHCs. Many commune health workers (CHWs) complained about having too many “supervisors.” While CHCs were technically supervised by the District Health Centres (for national target programs), by the District Centre of Family Planning and Population, and the District Hospital (for curative services), they also had to report to the District Health Bureau and the Commune People’s Committee that recruits and pays staff. No sooner than Circular 03 had been issued in 2008, CHCs were transferred to District Health Centres in Bac Giang in 2009, and in Lao Cai in 2012.

Participants in this study suggested that the district health reforms had shortcomings that resulted in asynchronous, ineffective and inconsistent management of CHCs. A district manager talked about the current organisation of district health in her province.

*“The fact is that our system is not good. Our current management is disconnected and inconsistent. I think health activities should be centralised, that it needs to have a centralised direction. In the district there are too many district health entities that all supervise CHCs, which can lead to desultory command. That is inconsistent”* (District level, Manager 2).

In these provinces, while CHCs are supervised by the District Health Boards, the fact that the District Health Centres and District Hospitals assign tasks for CHCs, but are not the entities that pay salaries and do not have a voice in personnel decisions, was seen as problematic. It was considered difficult to assign workloads and manage the performance of CHWs.

*“Talking about the management system, I see some problems. The District Health Bureau is in charge of the personnel in CHCs, but the District Health Centre and District Hospital supervise them technically. One person pays the salary and others assign tasks; things are not synchronous. For example, I force you to do many things but I do not pay you, it sounds difficult in management practice”* (Provincial level, Adminstrator 5).

Participants from the District Hospital reported that it was relatively difficult to implement technical direction in CHCs. District Hospitals have less power over doctors in the CHCs (who work through a contract, with the permission of the District Health Centre or the District Health Bureau) and they rarely provide professional support for CHWs because they do not manage personnel. A manager of a District Hospital expressed their concern about the accountability of CHCs to the District Hospital:

*“The situation is like two or three nooses put on one head of the CHC. One person pushes them to work, and the other pays the salary. Our hospital is responsible for the provision of the budget for examination and treatment and drugs but in fact we do not manage them. So if some mistakes or losses happen, we find it difficult to deal with. This is an economic issue. The second one is a technical issue. CHWs are under the management of the District Health Bureau and we only provide them technical supervision”* (District level, Manager 12).

Similarly, while the District Health Centre in Lao Cai is responsible for the management, mentoring and implementation of national target programs, it was not directly responsible for managing CHCs. Any program it developed needed to get approval from the District Health Bureau.

It is difficult for District Hospitals to effectively provide support to the CHC level and District Health Centres also face difficulties in mobilizing medical doctors in prevention programs. The functioning of the CHCs therefore depended heavily on the administrative and managerial collaboration between of all these district level entities. Participants complained about this situation:

*“If we are talking about adequacy, it is not. We [CHC] are under the different supervisions, technically and administratively. So we imagine it as having 3 nooses on our head. It is very complex” (*Commune level, Manager 2).

*“No one likes to have many fathers coming to you with a stick and admonishing you at the same time. If all fathers collaborate well, the children living underneath are less miserable. I think having only one person supervising you and taking care of you is much better that having many supervisors. But we are afraid that in our situation, many people give direction but none of them can take care of us. Every man for himself. Only the CHC is left”* (Commune level, Manager 4).

In some cases, CHCs did not know how to respond since each “boss” had a different direction and “the bosses rarely sit together.” One participant noted that the inconsistency of instruction and work direction created difficulties for CHCs.

*“Each person that comes to inspect has their own instructions. This person instructs us to do one thing, the other may instruct us to do some other thing, and then we have to redo things. In general, it would be better if they could work together closely” (*Commune level, Manager 4).

Inconsistent instruction from district health supervisors led to CHWs being confused or, in some circumstances, mistrusting those who supervised their facility. This may result in CHWs having “low responsiveness” as part of a coping strategy to avoid redoing work.

It was also reported by district level participants that ineffective collaboration amongst district health entities often led to programs being sequenced at the same time and this was considered to create difficulties for CHCs in terms of workflow. For example, a family health planning program and an extended vaccination program may take place on the same day in CHCs.

“Having too many bosses at the same time” and complicated reporting systems due to ineffective collaboration between district health entities were issues repeatedly raised by commune level participants**.** The district health entities established a multiplicity of reporting requirements. It was commonly agreed that this paperwork contributed to the workload that CHCs had to shoulder despite being short-staffed.

*“We have to submit many kinds of reports. If the District Health Centre collaborates well with the District Health Bureau, CHCs do not have to send reports to the District Health Bureau. Monthly we have to submit reports to the District Health Bureau on personnel, medicine use and some other vertical programs. We have to report about examination and treatment for insured patients to the hospital, vertical programs to the District Health Centre, some state administration programs to the District Health Bureau, and about population and family planning programs to the Centre of Family Planning and Population. The regular operation needs to report to the Commune People’s Committee. At the same time, we have to make many reports” (*Commune level, Manager 4).

In addition to reporting to the District Health Bureau, the District Hospitals and the District Health Centres, CHCs also had considerable paper work for health insurance payment procedures. Writing reports was considered to take up a large proportion of the working hours of CHWs. Some respondents emphasised that there were dozens of reports, including weekly, monthly, quarterly, and annual reports that they needed to submit to three district health entities and other bodies. This definitely reduced the time they could spend on clinical work and therefore influenced their job performance.

In many districts, CHCs only submitted specific reports relevant to each supervisor, not a report about the whole operation of CHCs. For example, CHCs reported only examination and treatment activities to the District Hospital, and the implementation of vertical programs to the District Health Centre. Therefore, the three supervisors would not grasp the whole operation of CHCs if they did not collaborate closely.

## Discussion

**Figure 3:**
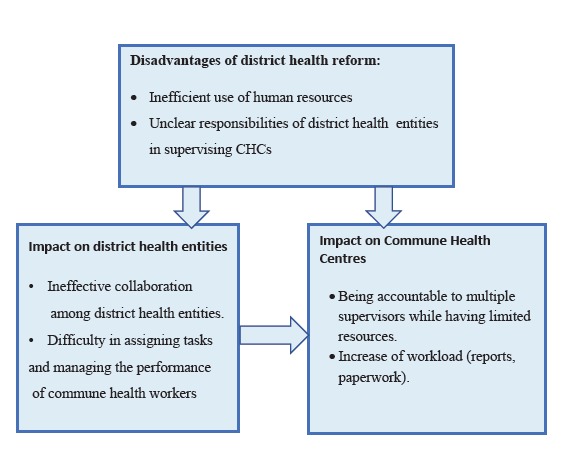
Influence of District Health System Reforms on Organisation of Service Delivery.

This study has shown that while the reforms were premised on the belief that there would be gains associated with the separation of functions, the changes have also resulted in a range of dysfunctionalities that negatively impact on the effective governance of district and commune level health services. This is illustrated in Figure 3.

In summary, the three district health entities operate independently, each focusing on different but overlapping issues. There was a clear gap in the management of maternal health service delivery, particularly the quality of supervision of maternal health services delivery provided at the CHCs. The management of health services delivery at the CHCs requires integration between the administrative and technical management as well as direction in the curative and preventive medicine areas.

Although each country’s reform process has its own specificity, there are common trends to achieve national health goals and objectives, such as the pursuit of better health outcomes in terms of quantity and quality of services, and more efficient use of resources. Particularly in the health sector, these objectives are commonly expressed in terms of improving equity of access to services, effectiveness of care, efficient utilization of resources, satisfaction of users, and sustainability.^31^ The health sector reforms which have been put in place in many countries have typically involved decentralization,^32^ privatization,^12^ managerialism, separation of entities (insurers and providers),^10^ and the separation of curative and preventive services.^33^

This research suggests that fragmentation in the Vietnamese health system occurred when reforms to district level governance saw a move from an integrated model to a model where functions were disaggregated. This disaggregation resulted in a lack of coordination between entities at the district level, inefficiencies in terms of the costs (financial and other) of developing and maintaining multiple management structures at the district level^10^ and consequences for the management and service delivery responsibilities of the CHCs which post-reform have multiple accountability relationships.^34^ These findings are consistent with earlier reports from Vietnam that noted that the division of district health services into three units caused disconnection between the different functions of the health system and caused confusion within CHCs.^16,35^

The World Health Organization (WHO) noted that fragmentation can cause negative consequences for the efficiency of the system as a whole.^36^ Previous studies have identified that the separation of vertical programs, such as Tuberculosis, HIV/AIDs prevention, and childhood illness,^37^ has led to fragmentation. Previous research has also noted that the separation of curative and preventative health programs may impact the coordination of primary care in developing countries.^37^ A systematic review of decentralization of health systems in low and middle income countries indicated that some countries experienced coordination problems between central and local levels.^32,37^ The negative consequences of fragmentation can include fiscal inefficiencies associated with additional costs of management but also the costs associated with poor coordination of activities. Previous research has noted that “fragmentation by itself or in conjunction with other factors can lead to difficulties in access to services, delivery of services of poor technical quality, irrational and inefficient use of resources, unnecessary increases in production costs, and low user satisfaction.”^13^ Participants in this research expressed concerns about resource use, and service delivery.

The impact of this fragmentation was particularly problematic in respect of the effective operating of CHCs, which provide primary healthcare at a local level, as the operational guidance and supervision was reported to be somewhat loose and accountabilities were unclear. This was said to have led to a reduction in operational efficiency of CHCs. The lack of clarity in respect of supervision raised operational problems about what directives to follow and when. Accordingly, CHWs may not be appropriately supervised in all areas of their practice and made it more likely that some issues critical to the delivery of quality of care may not be adequately supervised. This result mirrors the findings from research conducted in Cambodia.^38^ Fragmentation at the district level also resulted in multiple layers of approval processes and multiple accountability requirements leading to a significant burden being imposed upon CHCs in terms of reporting and documentation requirements. This result mirrors results in previous desk review articles from developing countries.^39^ These unclear accountabilities may have contributed to fragmentation of the understanding of what was needed for truly integrated care provision at the local level, as in some cases no one entity at the district level could see the whole picture in terms of the CHCs operations. That several participants used the imagery of a hangman’s noose to describe how they felt about the supervision they received from the district level entities and the apparent lack of coordination says much about the impact of this fragmentation of supervision on the morale of CHWs and their level of trust in the governance framework within which they operated.

It is evident that the Vietnamese reforms have had an impact on the health workforce. The creation and maintenance of three entities at the district level was reported as an inefficient use of human and other resources, as managers for each entity were required. The managers of these entities came from senior clinical staff and this had the effect of either limiting the time those persons could spend on clinical care or requiring them to work effectively two jobs in a rural context where it was already difficult to recruit and retain staff.^40^ Additionally, these managers did not have the management expertise to effectively manage their staff which in turn lead to criticism by staff of their lack of management background and skills.^23^ Recent research in Vietnam showed that, poor supervision from managers was perceived as demotivating for health workers in rural areas.^41^ This situation is not restricted to Vietnam. In general in developing countries, local level health managers are often found to lack managerial skills,^4,33,42^ but this may be exacerbated in Vietnam given the design of the district health system. Moreover, the transfer of personnel administration and budget has negatively influenced the working conditions and motivation of health workers which has also been seen in previous research in other countries.^31^

## Limitations

The study provinces and districts were purposively not randomly sampled for practical and resource reasons. We believe this sample provides insights into what is happening across Vietnam. However, as demonstrated there is considerably variability in the implementation of the reforms so results may not be generalizable to all provinces.

## Conclusion

Internationally, there has been a movement towards the development of integrated systems at local levels to enable the provision of carefully coordinated and comprehensive primary care services to improve health outcomes.^13,14,37,43,44^ This research indicates some of the problems from a governance perspective when services at the district level are not integrated and the unintended consequences that health system reforms can have on the provision of clinical services.

The quality of maternal healthcare is influenced by the structure and informal practices of the health system within which such services are offered.^45^ This analysis raises questions about the efficiency and overall utility of the separation of administrative, curative, preventative and family planning related functions at the district health level as implemented in Vietnam. If such separation of curative and preventative functions is considered desirable, reformers also need to devote attention to developing governance mechanisms that clarify the responsibilities of each entity. These must be clearly delineated in policy and be clearly understood by those who work within those entities and by the organisations and individuals that those entities supervise. The requirement for effective coordination must also be clearly delineated. Mechanisms should be put in place to ensure that such coordination occurs so as to maximise health outcomes, to, as much as possible, assure efficiencies both in terms of operations and costs and to lessen confusion and uncertainty experienced by health workers.

## Acknowledgements

We are grateful to the study participants who agreed to be interviewed and provide information related to district health reforms and maternal healthcare services.

## Ethical issues

Research ethics approval was obtained from the Queensland University of Technology, Brisbane, QLD, Australia research ethics committee (approval number 1200000087). Informed consent was obtained from all participants and they were informed of their right to withdraw.

## Competing interests

Authors declare that they have no competing interests.

## Authors’ contributions

All authors contributed to the study design. NTHT conducted data collection and analysis. NTHT, FM, and SW prepared the first draft of this paper. AW, SW, and FM contributed and commented on the draft. All authors read and approved the final manuscript.

## Authors’ affiliations

^1^Institute for Preventive Medicine and Public Health, Hanoi Medical University, Hanoi, Vietnam. ^2^Australian Centre for Health Law Research, Faculty of Law, Queensland University of Technology, Brisbane, QLD, Australia. ^3^Institute for Global Health and Development, Queen Margaret University, Musselburgh, UK. ^4^Menzies Centre for Health Policy, University of Sydney, Sydney, NSW, Australia.

## Key messages

**Implications for policy makers**

- The district health reforms in Vietnam caused disconnection between the different functions of the health system, and inefficiencies in terms of the costs of developing and maintaining multiple management structures at the district level.
- The division of district health services into three units exacerbated the existing shortages of qualified health workers in some areas.
- If the separation of curative and preventative functions is considered to be desirable, attention needs to be paid to developing governance mechanisms that clarify roles and responsibilities and deliver coordination so as to maximise health outcomes.

**Implications for the public**

This research suggests that fragmentation in Vietnam occurred when reforms to district level governance saw a move from an integrated model to a model where functions were disaggregated. This disaggregation resulted in a lack of coordination between entities at the district level, and inefficiencies in terms of the costs (financial and otherwise) of developing and maintaining multiple management structures at the district level. This imposed further costs on the commune health centres (CHCs), which have multiple accountability relationships. If the separation of curative and preventative functions is considered to be desirable, this research demonstrates that attention needs to be paid to instituting mechanisms that ensure clear responsibility and effective coordination. In other words, the responsibilities of each entity must be clearly delineated in policy and be clearly understood by those who work within those entities.
